# Transmission probability filter optimization for Agility MLC in Monaco treatment planning system

**DOI:** 10.1002/acm2.14105

**Published:** 2023-07-26

**Authors:** Lena Thewes, Miriam Eckl, Frank Schneider

**Affiliations:** ^1^ Department of Radiation Oncology University Medical Centre Mannheim University of Heidelberg Mannheim Germany

**Keywords:** MLC transmission in Monaco TPS, transmission probability filters

## Abstract

In the Monte Carlo‐based treatment planning system (TPS) Monaco, transmission probability filters (TPF) are utilized to describe the transmission through the multi leaf collimator (MLC). By having knowledge of the TPF parameters for various photon beam energies, adjusting the MLC transmission parameters becomes easier, enhancing the accuracy of the Monte Carlo algorithm in achieving a dose distribution that closely aligns with the irradiated dose at the Versa HD linear accelerator (linac). The objective of this study was to determine the TPF parameters for 6MV, 10MV, 6MV flattening filter free (FFF) and 10MV FFF for a Versa HD linac equipped with Agility MLC. The TPF parameters were adjusted using point dose measurements and vendor‐provided fields specifically designed to fine‐tune the MLC. After adjusting the TPF parameters, a gamma passing rate (GPR) analysis was conducted on 25 treatment plans to ensure that the Monte Carlo model, with the updated TPF parameters, accurately matched the actual linac delivery. The TPF values ranged from 0.0018 to 0.0032 for leaf transmission and 1.15 to 1.25 for Leaf Tip leakage across the different energies. The average GPR ranged from 97.8% for 10MV FFF to 98.5% for 6MV photon energies. Additionally, the TPF parameters for 6MV obtained in this study were consistent with previously published TPF values for 6MV photon energy. Hence, it was concluded that optimizing the TPF does not need to be performed for every individual Versa HD linac with Agility MLC. Instead, the published parameters can be applied to other Versa HD linacs to enhance clinical accuracy. In conclusion, this study determined the TPF parameters for 6MV and previously unpublished photon energies 10MV, 6MV FFF and 10MV FFF. These parameters can be easily transferred to other facilities, resulting in improved agreement between the dose distribution from the TPS and the linac.

## 1 INTRODUCTION

In intensity modulated radiation therapy (IMRT), having a treatment planning system (TPS) that accurately models the linear accelerator beam is crucial for the entire treatment workflow. It enables precise simulation of mechanical treatment plan delivery and dose deposition in the patient at sub‐millimeter precision. Therefore, it is essential for the multi leaf collimator (MLC) transmission parameters to precisely match the setup of the linear accelerator (linac) to ensure an accurate simulation of the irradiated dose to the patient.^1^ The TPS Monaco (Elekta AB, Stockholm, Sweden) utilizes the X‐ray voxel Monte Carlo (XVMC) dose calculation algorithm. Within this algorithm, the user can adjust the MLC transmission parameters by customizing the transmission probability filters (TPF) in the TPS for each energy. The TPFs include both geometrical parameters and transmission parameters. The most relevant geometrical parameters are an offset to the default leaf position and the distance between two adjacent leaves. The transmission parameters mainly include the transmission through the leaf or jaw body, the leakage between adjacent leaves and the leakage through the leaf tip. These TPFs model the probability of a photon transmission through the MLC.^2^, ^3^, ^4^ Previous studies^1^, ^4^ described how these TPF parameters can be adjusted on Elekta Versa HD (Elekta, Crawley, UK) or Elekta Synergy (Elekta, Crawley, UK) linacs to achieve an accurate representation of the linac head. This adjustment is performed using ionization chamber measurements and vendor‐provided field known as ExpressQA beams, which are designed for MLC adjustment. This expands upon the recommended measurements during the linac and TPS commissioning, which typically include dose profiles, depth dose curves and some sliding window field measurements.^5^, ^6^ However, this extended commissioning process is essential because if the TPF parameters are not selected correctly in the TPS, it can lead to calculated doses higher than the irradiated dose in the IMRT plans for example. This can occur due to too large field sizes or high transmission caused by the chosen TPF parameters in the TPS. As a result, the target volume may receive an inadequate, too low dose during radiation. In the same way, incorrectly selected TPF parameters can also result in a too high dose in the organs at risk. To avoid such under‐ or overdose, it is crucial to select the TPF parameters correctly. To our knowledge, only TPF parameters for 6MV photon energy have been published thus far. However, TPF parameters for other energies are also of interest, as establishing robust and accurate TPF parameters for multiple photon beam energies facilitates reliable adjustment of the MLC transmission parameters for every linac of the same model. With knowledge of the TPF parameters, TPS commissioning can be substantially accelerated, even with limited user experience, as only the parameters need to be considered, a single measurement for verification is required, and if this measurement fits no further adjustments are necessary to achieve a precisely modeled linac within the respective TPS.

The objective of this study was to determine the Monaco TPF parameters for the MLC transmission at several energies used for IMRT on a Versa HD linac with Agility MLC and Elekta Accelerated Go Live (AGL) standard beam model. The analysis included photon energies of 6MV with flattening filter (FF), 10MV FF and 6MV and 10MV without flattening filter (FFF). Furthermore, the TPF parameters for 6MV FF energy were compared to previously published values to validate their robustness for different unmatched linacs of the same model.^4^

## 2 MATERIALS AND METHODS

Adjustment of the TPF parameters was carried out on the XVMC model within the TPS Monaco (Version 6.1.1), which was used as the TPS for two Versa HD linacs equipped with Agility MLC and AGL beam model. The analysis included photon energies of 6MV, 10MV, 6MV FFF and 10MV FFF. Table 1 presents the various editable parameters available for adjusting the TPF.^7^ All of these parameters were used for editing the default TPF.

**TABLE 1:** Manufacturer and obtained transmission probability filter parameters of the multi leaf collimator transmission parameters for the different energies fitting for both tested Versa HD linacs.

	6MV	10MV	6MV FFF	10MV FFF
Leaf transmission	0.0032	0.0029	0.0022	0.0018
Interleaf leakage	7	6.34	4.81	3.94
Leave Groove Width (mm)	0.4	0.4	0.4	0.4
TJaw transmission	0.0042	0.0041	0.003	0.0027
LeafTip leakage	1.18	1.23	1.15	1.25
Leaf offset (mm)	0	0	0	0

### 2.1 Adjustment of the TPF parameters for 6MV, 10MV, 6MV FFF and 10MV FFF

The optimization of the MLC parameters followed a procedure similar to recommended approaches outlined in previous studies.^1^, ^4^ Initially, default values were selected for all TPF parameters. Then, each parameter was iteratively adjusted until it matched the tolerances of the measurements performed at the linac, as described below.

First, the leaf transmission and interleaf leakage was measured using a farmer chamber (Type 30001, PTW dosimetry, Freiburg, Germany) and a rigid stem chamber (Type 23332, PTW dosimetry, Freiburg, Germany) in a 30 × 30 × 30 cm^3^ water phantom. The chambers were positioned at the isocenter, with a source‐to‐surface distance (SSD) 100 cm and in a depth of 5 cm. A field with a 0.5 mm wide gap, located 15 cm away from the center and utilizing the full 40 cm length of the MLC with maximum opened Y‐jaws, was used. The dose was measured five times under the closed leaves and compared to the dose calculated in the chamber volume contoured in the TPS using the same measurement setup. The TPF parameters were manually adjusted in in steps of 0.0001 for the leaf transmission parameter and 0.2 for the coarse and 0.01 for the fine adjustment of the interleaf leakage parameter. Iterations of these adjustments were performed until the dose value in the TPS in the previously defined ionization chamber volume in the water phantom matched the average of the measured data within an accuracy of 0.1cGy, which is the standard deviation.

Next, the Y‐jaw transmission was measured five times using the same ionization chambers and setup as for the leaf transmission measurement. A field with a 0.5 mm wide jaw gap, located 15 cm away from the center, while the MLCs were fully opened, was used. The dose in the center under the closed jaw was measured, and the TJaw transmission parameter was iteratively adjusted until the dose value calculated in the contoured chamber in the TPS matched the measured values for the different energies with a maximum deviation of 0.1cGy. The TJaw transmission value was determined with an accuracy of 0.0001.

**FIGURE 1:**
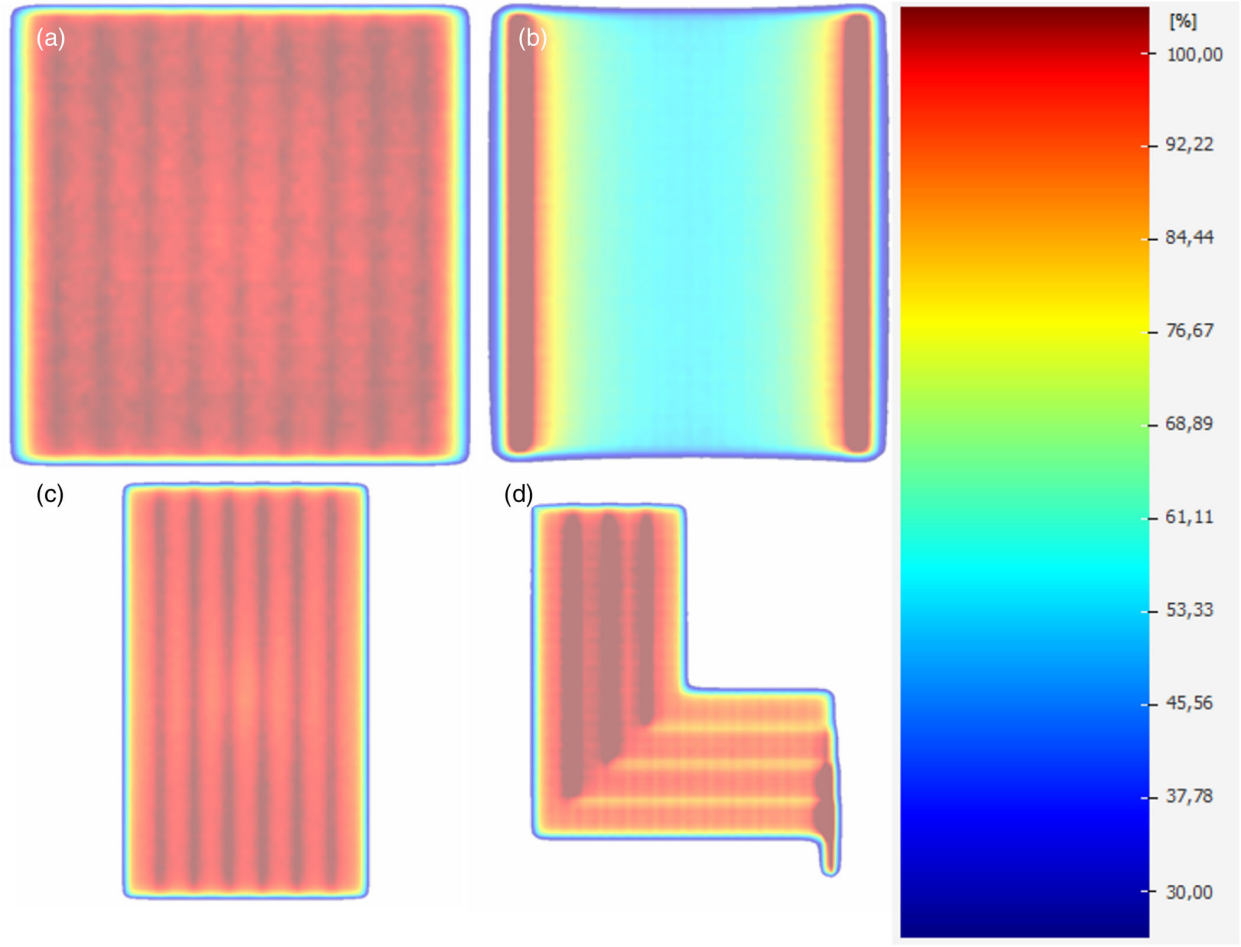
Dose distributions of one plane of four of the ExpressQA beams used to adjust the TPF parameters for leaf offset and leaf tip leakage with (a) Dynamic sweeping gap representing the movement of a 1 cm–2 cm slid over a 20 × 20 cm field, (b) Plan with a rotating gantry representing the movement of a 1 cm slid over a 20 × 20 cm field with an off‐axis dose maximum, (c) Step‐and‐shoot field with seven 2 cm wide MLC slits, (d) Step‐and‐shoot plan with four L‐fields. MLC, multi leaf collimator; TPF, transmission probability filters.

In the third step, the leaf offset was determined by evaluating the field size in a dose profile measurement of a 30 × 30cm^2^ field in a water phantom (Blue Phantom 2, iba dosimetry, Schwarzenbruck, Germany) with an SSD 90 cm in 10 cm depth. This measurement was repeated 4 times. This field size at 50% between maximum and minimum dose was compared to the field size calculated in the TPS using a water box of the same size as the water phantom using different leaf offsets. The leaf offset was iteratively optimized in steps of 0.05 mm until the smallest deviation to the measured field size was detected. Furthermore, the leaf offset was validated by analyzing ExpressQA beams, which are recommended by the vendor to adjust the MLC.^1^, ^7^ Two plans were considered for the validation. First, a plan based on a dynamic sweeping gap with a field size of 2 cm depicted in Figure 1a) was regarded. This plan should result in a flat and homogeneous dose profile using a FF beam if the leaf offset parameters were chosen correctly. Second, a step‐and‐shoot field with seven 2 cm wide fields, shown in Figure 1c), was examined. The junctions were used to analyze the fitting field size and leaf offset. For both plans it was important to ensure that the profile is flat, homogeneous, and best matched the linac using the leaf offset parameter chosen based on the field size measurement.

The leaf groove width was adjusted as described by Roche et al.^4^ The vendor‐provided ExpressQA beams visible in Figure 1 were used for this purpose. No other values were recommended due to the limited ability of the TPF parameters to accurately model the groove.

The final step of the MLC modelling consisted of the adjustment of the leaf tip leakage. This adjustment was performed using the ExpressQA beams, including the plan with a dynamic sweeping gap, a step‐and‐shoot plan with four L‐fields, the step‐and‐shoot field with seven 2 cm wide fields and a plan with a rotating gantry and a sweeping gap delivering the maximum dose outside of the center (displayed in Figure 1). The leaf tip leakage was modeled based on four measurements per energy, ensuring that the calculated ExpressQA beam fields matched the measurements as closely as possible. Adjustments in the leaf tip leakage were made in steps of 0.01 and were necessary if the calculated dose in Monaco exhibited deviations larger than ± 0.5% compared to the measured dose, considering the dose profile. The measurements were performed using the 2D ionization chamber array Matrixx (iba dosimetry, Schwarzenbruck, Germany) placed in the gantry holder at a source‐detector‐distance of 100 cm using a manually configured calibration per energy.

Subsequent to the ExpressQA beam analysis and based on the adjusted MLC transmission parameters, 25 VMAT patient plans per energy were calculated in the TPS in the Matrixx geometry for further QA measurements. These patient plans covered various disease sites and were measured at the two Versa HD linacs using the 2D‐detector Matrixx. A comparison of the measured dose with the calculated dose in the TPS was performed using the gamma passing rate (GPR) analysis. The GPR comparison aimed to ensure that the adjusted MLC transmission parameters fit well with the patient plans and could be effectively used in clinical routine. The tolerances for the GPR analysis were set at 2% dose tolerance and 2 mm spatial tolerance, with a dose cut‐off at 10% and a global criterion. These tolerances were smaller than the ones recommended for patient‐specific quality assurance (QA) but were suitable for modeling the MLC in the TPS.^1^, ^4^, ^8^

### 2.2 TPF parameters for different linacs of the same model

Additionally, it was relevant to evaluate whether the determined TPF parameters were identical for all Versa HD linacs with Agility MLC. A comparison was made between the TPF parameters obtained in this work and the parameters determined by Roche et al.^4^ for the 6MV FF energy (referred to as Model A). Furthermore, the 25 patient plans used in Chapter 2.1 were recalculated using the TPF values based on Model A, and GPR calculations were performed to analyze the agreement between the measured and calculated doses.

## 3 RESULTS

### 3.1 Adjustment of the TPF parameters for 6MV, 10MV, 6MV FFF and 10MV FFF

In Table 1, the results of the MLC transmission parameters for 6MV, 10MV, 6MV FFF, and 10MV FFF, which were found to fit adequately for both Versa HD linacs considered in this study, are presented.

**FIGURE 2:**
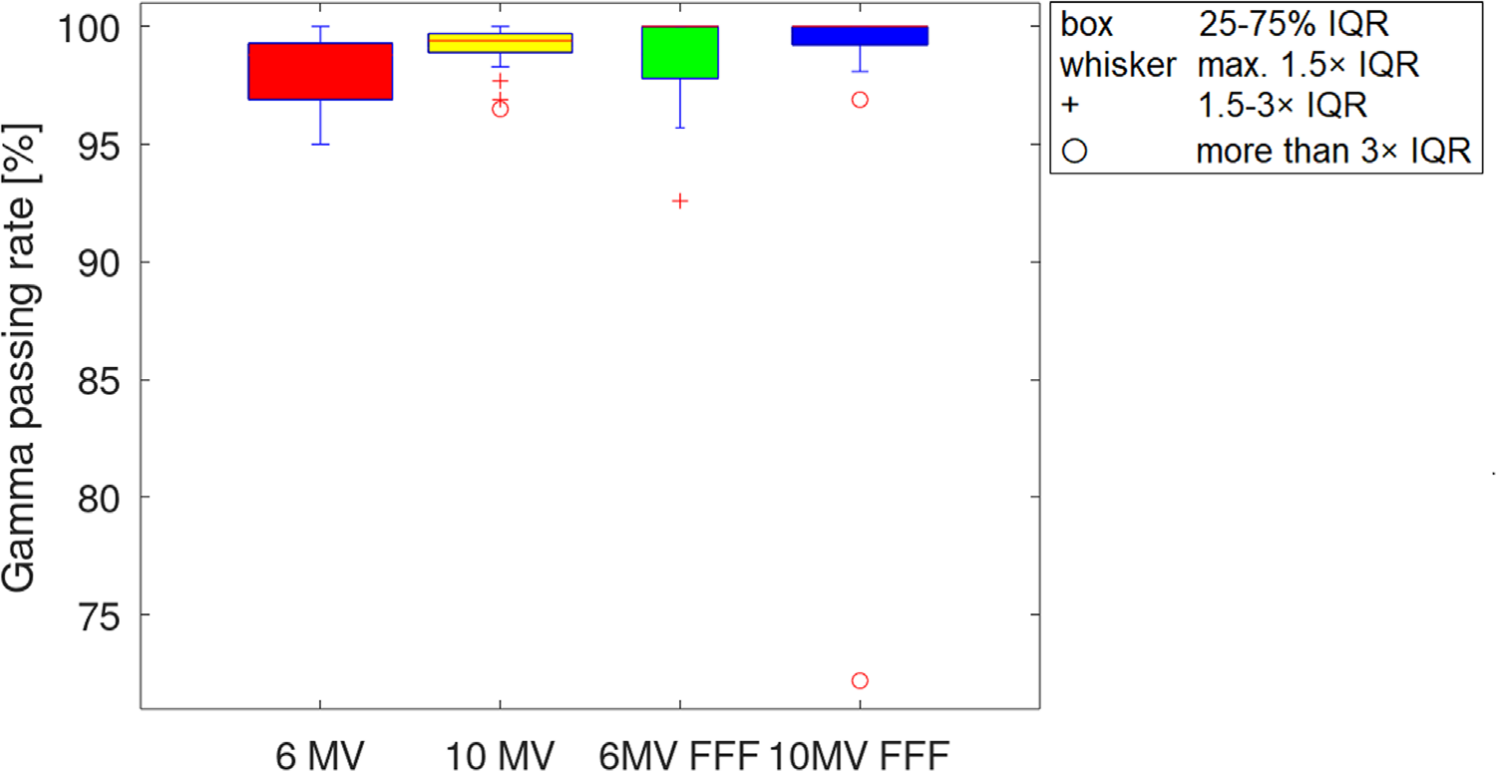
GPRs for 25 measured patient plans per energy using the adjusted TPF parameters visible in red for 6MV, yellow for 10MV, green for 6MV FFF and blue for 10MV FFF. GPR, gamma passing rate; TPF, transmission probability filter.

Regarding the 6MV energy, the GPR for the 25 measured patient plans was 98.4% ± 1.5% (range 95.0%–100%) using the TPF parameters adjusted in this study. The mean GPR for 10MV energy was 98.5% ± 2.0% (range 91.0%–100%). For the 6MV FFF energy the mean GPR was 98.2% ± 2.6% (range 92.6%–100%) and the 10MV FFF energy resulted in a mean GPR of 97.8% ± 6.9% (range 72.2%–100%). The results are graphically presented in Figure 2.

### 3.2 TPF parameters for different linacs of the same model

In comparison to the Model A,^4^ there are two TPF parameters that differ in this study, namely TJaw transmission and Leaf Offset. Roche et al.^4^ found a TJaw Transmission parameter of 0.0032, whereas in this study a parameter of 0.0042 was obtained. When using the parameter of 0.0032 in this study, the measured dose under the closed jaw was 9% higher than the dose calculated in the TPS. However, when comparing the GPRs of the 25 measured patient plans with the different TJaw transmission parameter but the same leaf offset of 0 mm, the mean value was 98.4% for both TJaw transmission values. This suggests that, on average, there was no difference, and the largest difference observed between the measured patient plans with different TJaw transmission values was 0.9%.

**FIGURE 3:**
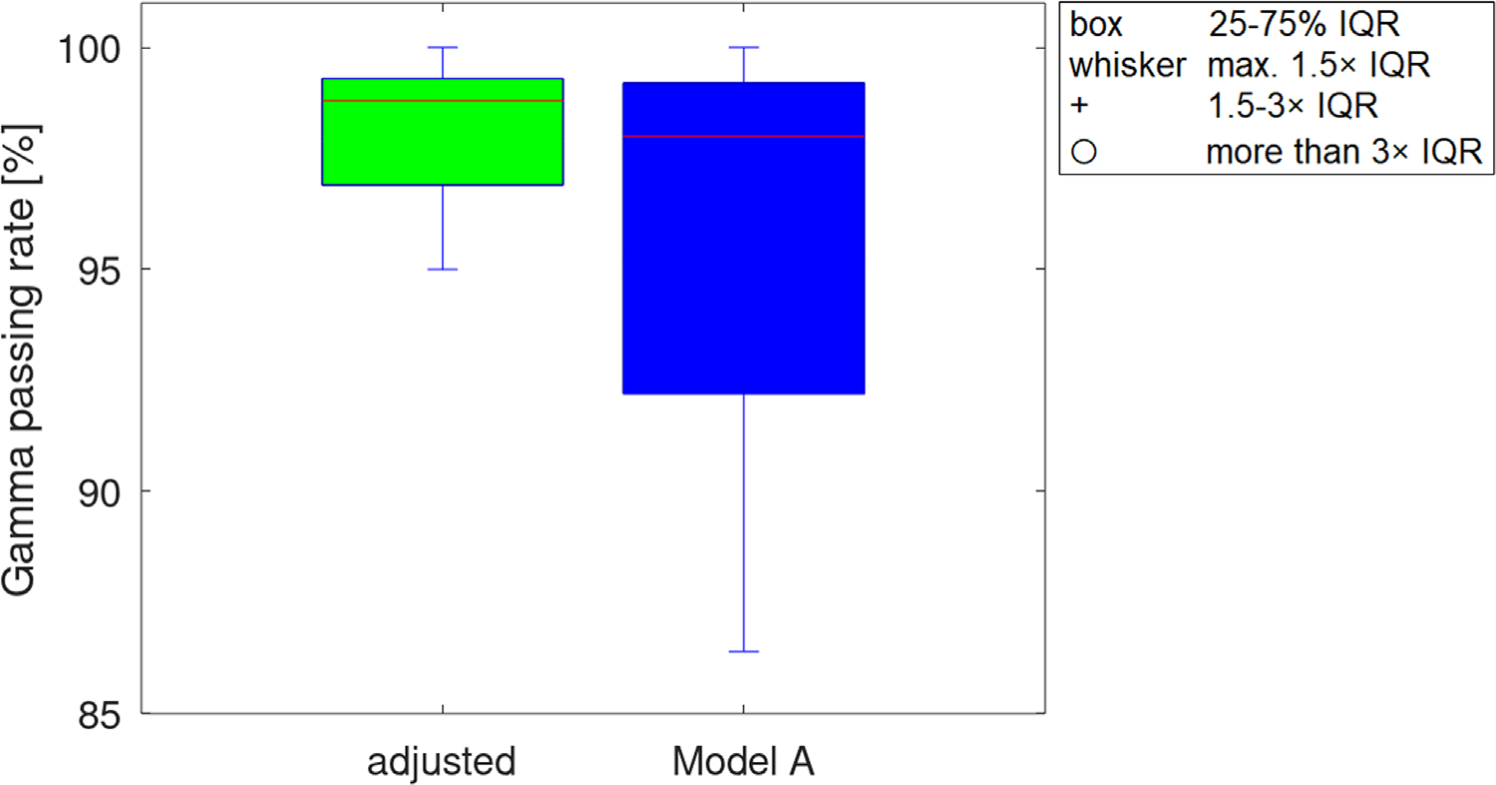
The GPR of the Model A is lower than the respective GPR of the model investigated in this study. GPR, gamma passing rate.

Regarding the Leaf Offset, Roche et al.^4^ detected a value of −0.05 mm, while in this study, a Leaf Offset of 0 mm was determined for a field size that matched the water phantom measurement and the sweeping gap field. When using the Leaf Offset and the TJaw transmission value from Model A, the mean GPR compared to the measurement of the 25 6MV patient plans of 96.0% ± 3.9%. In comparison, as mentioned in Chapter 3.1, the mean GPR with the adjusted parameters was 98.4% ± 1.5%. The differences are graphically presented in Figure 3.

## 4 DISCUSSION

To have a dataset of MLC transmission parameters that are different from the default values but fit well with most Versa HD linear accelerators, makes the commissioning of the TPS much easier. Once these MLC transmission parameters are known, they only need to be measured once for verification. This leads to a reduction of the repetitive adjustment by the user, because the TPF parameters can simply be changed to the published values. This approach leads to improved clinical results compared to using the default values. Such MLC transmission parameters were identified in this study for four different photon energies.

### 4.1 Adjustment of the TPF parameters for 6MV, 10MV, 6MV FFF and 10MV FFF

The adjustment of TPF parameters for the photon energies 6MV, 10MV, 6MV FFF and 10MV FFF was shown to fit two investigated Versa HD linacs. Based on the presented TPF parameters, another time‐consuming step within the process of MLC modelling can be substantially facilitated because it eliminates the need for users to determine the TPF parameters themselves through repetitive adjustments. Instead, users can directly apply the published TPF parameter values and only need to verify them, resulting in accurate clinical dose distribution. Because only two Versa HD linacs were available to test this assumption, a check whether the parameters only need to be verified by one measurement instead of performing several repetitions to adjust the TPF with more Versa HD linacs would be useful.

In this work, to measure the leaf transmission and interleaf leakage separately, various methods were attempted. Initially, both were measured together using a 0.6 cm^3^ farmer chamber. Then, an attempt was made to measure leaf transmission using a 0.015 cm^3^ pin point chamber positioned under a closed leaf, but not under a leaf gap. However, the measured leaf transmission values were consistently higher than the combined value of leaf transmission and interleaf leakage, which is physically impossible. As a result, the factor between leaf transmission and interleaf leakage as detected by Roche et al.^4^ was used without further modifications. However, as mentioned by Snyder et al.,^9^ some of the MLC parameters influence and compensate each other. Thus different leaf transmission and interleaf leakage values can compensate for each other and therefore lead to the same dose. However, if this dose is correct, it can be assumed that the TPF values for the leaf transmission and interleaf leakage can be reliably applied.

Additionally, a modified groove width value was evaluated, but no visible change in the ExpressQA beam fields was detected. Therefore, the value described by Roche et al.^4^ was chosen.

The use of a 2D ionization chamber array to measure the ExpressQA beam fields resulted in low resolution due to the detector's chamber spacing of 7 mm. However, agreements were found between the measured values at the locations where the detector's measuring points were located. This suggests that even with this low resolution, the ExpressQA beam fields could be adjusted based on the detector measurements. Nonetheless, the low resolution of the detector led to occasional outlier plans when comparing the 25 measured plans per energy to the TPS. Particularly in patient plans with a diameter of around 2 cm with large dose gradients, the detector resolution was insufficient, resulting in outliers that did not fulfill the 95% gamma passing criteria.

Previous studies, such as Snyder et al.,^9^ have also demonstrated that the MLC parameters provided by the vendor may not optimally fit the linac, particularly in point dose measurements. This finding is consistent with the observed effects of leaf transmission in this study. The importance of accurate MLC modeling in Monte Carlo simulations of the linac head has been emphasized in various studies.^1^, ^4^, ^9^, ^10^ However, none of the mentioned studies analyzed the TPF parameters required for energies other than 6MV, which is a contribution of this study.

### 4.2 TPF parameters for different linacs of the same model

Adopting the TPF parameters detected in this study for other Versa HD linacs only makes sense if it can be assumed that linacs from the same model can also use the same TPF parameters. Therefore, this was evaluated by comparing the detected TPF parameters with those published by Roche et al.^4^ for the 6MV FF energy.

As mentioned in Chapter 3.2, one discrepancy between the detected TPF parameters in this study and those published by Roche et al.^4^ was found in the TJaw transmission value. However, despite the difference, it was observed that the GPRs remained unaffected. This indicates that the TJaw transmission has minimal influence on the GPR. Consequently, even if the TJaw transmission values deviate between the two studies, both values can still be used effectively for accurate dose calculations in the TPS.

Regarding the leaf offset, it was observed that a value of −0.05 mm, as chosen by Roche et al.,^4^ was not necessary for the linacs used in this study. In fact, using a leaf offset of 0 mm resulted in TPS field sizes that were 0.1 mm too small compared to the measurements. With a leaf offset of −0.05 mm the field size in the TPS became smaller, with +0.05 mm the field size became too large compared to the measurements. For this reason 0 mm leaf offset was chosen. Additionally, regarding the ExpressQA beams, using the leaf offset of 0 mm resulted in less cold spots than with −0.05 mm leaf offset and an improved dose profile homogeneity. Furthermore, it was assumed that a systematic leaf offset would correspond to an inaccurate field size either in Monaco or at the linac. With correctly functioning systems, no leaf offset should be required and the value can be set to 0 mm. Another reason for this difference could be found in the usage of different head models. In this study, the Elekta AGL standard beam model was used while Roche et al.^4^ used a head model based on base data measurements, which could explain other minor discrepancies in TPF parameters found in this study as well. By using the AGL standard beam model, it is believed that the TPF parameters identified in this study can be transferred to centers setting up their Versa HD linacs and Monaco TPS within the AGL program.

Apart from the leaf offset and TJaw transmission, all other parameters for the 6MV FF photon energy were identical to those reported by Roche et al.^4^ Therefore, it can be assumed that the TPF parameters are generally valid for Versa HD linacs with Agility MLC and the TPF parameters found in this study for the 4 different photon energies can therefore be used for every linac of this model, at least to be used as a good starting point for further fine tuning.

## 5 CONCLUSION

The optimization of MLC transmission parameters for 6MV, 10MV, 6MV FFF, and 10MV FFF energies in this study resulted in improved agreement between the dose distribution calculated by the TPS and the dose delivered by the linac compared to the default parameters. This improvement was validated by achieving a substantially better GPR, indicating a closer match between the calculated and delivered dose. Besides, it was demonstrated that the TPF parameters adjusted for one Versa HD linac are equal to TPF parameters. This finding suggests that the TPF parameters obtained in this study can be transferred to other centers that are setting up Versa HD linacs and using Monaco TPS within the AGL program. Instead of performing multiple repetitions to adjust the TPF parameters for each individual linac, these parameters only need to be measured once for verification, and small adjustments can be made if necessary.

## AUTHOR CONTRIBUTIONS

MLC transmission in Monaco treatment planning system. L. Thewes adjusted the transmission probability filters. L. Thewes wrote the manuscript with support from M. Eckl and F. Schneider.

## CONFLICTS OF INTEREST STATEMENT

No conflicts of interest.
